# Intentional binding effect in children: insights from a new paradigm

**DOI:** 10.3389/fnhum.2014.00651

**Published:** 2014-08-25

**Authors:** Annachiara Cavazzana, Chiara Begliomini, Patrizia S. Bisiacchi

**Affiliations:** ^1^Department of General Psychology, University of PaduaPadova, Italy; ^2^Center for Cognitive Neuroscience, University of PaduaPadova, Italy

**Keywords:** sense of agency, intentional binding, voluntary action, development, frontal lobe

## Abstract

Intentional binding (IB) refers to the temporal attraction between a voluntary action and its sensory consequence. Since its discovery in 2002, it has been considered to be a valid implicit measure of sense of agency (SoA), since it only occurs in the context of voluntary actions. The vast majority of studies considering IB have recruited young adults as participants, while neglecting possible age-related differences. The aim of the present work is to study the development of IB in 10-year-old children. In place of Libet’s classical clock method, we decided to implement a new and more suitable paradigm in order to study IB, since children could have some difficulties in dealing with reading clocks. A stream of unpredictable letters was therefore used: participants had to remember which letter was on the screen when they made a voluntary action, heard a sound, or felt their right index finger moved down passively. In Experiment I, a group of young adults was tested in order to replicate the IB effect with this new paradigm. In Experiment II, the same paradigm was then administered to children in order to investigate whether such an effect has already emerged at this age. The data from Experiment I showed the presence of the IB effect in adults. However, Experiment II demonstrated a clear reduction of IB. The comparison of the two groups revealed that the young adult group differed from the children, showing a significantly stronger linkage between actions and their consequences. The results indicate a developmental trend in the IB effect. This finding is discussed in light of the maturation process of the frontal cortical network.

## Introduction

The feeling of generating and controlling actions and their external effects is known as sense of agency (SoA; Haggard and Tsakiris, 2009). When we act, we are generally in control of what we are doing; therefore, we are aware and responsible for both our own actions and their consequences.

For many years, researchers have tried to identify appropriate measures to study this complex phenomenon. Two main research lines can be distinguished (Synofzik et al., 2008). The first refers to agency at its explicit level: usually, explicit agency is investigated by tasks in which participants have to verbally report whether they feel they are the authors of a certain effect or whether a presented sensory feedback of an action corresponds to the action made (Wegner and Wheatley, 1999; Aarts et al., 2005; Sato and Yasuda, 2005; Daprati et al., 2007; Metcalfe and Greene, 2007; Tsakiris et al., 2007; Farrer et al., 2008). However, we experience a continuous flow of actions and their effects in our everyday life, and we do know that we are the authors of an action without constantly giving explicit judgments. The second research line on SoA involves implicit measures, such as intentional binding (IB; Haggard et al., 2002). Such an effect occurs when a temporal compression phenomenon between voluntary action and its consequent effect is observed (e.g., actions are perceived as occurring later than they really do, while the sensory effect is perceived as occurring earlier). This effect seems to be limited to voluntary actions; in fact, IB is absent or reduced for situations in which the action is not driven by volition (e.g., passively-induced movement) or when no intentional agent is present (Haggard et al., 2002; Haggard and Clark, 2003; Engbert et al., 2008). Since its discovery, IB has been considered a valid quantitative index of SoA and has been applied to study agency, both in healthy individuals and clinical populations (for a review, see Moore and Obhi, 2012).

Up to the present moment, studies on SoA in general—and IB in particular—have concentrated most of their attention on searching for the underlying cognitive and neural mechanisms (David et al., 2008; Moore et al., 2010; David, 2012; Moore and Obhi, 2012; Kühn et al., 2013; Jo et al., 2014), without considering the aspect of ontogenetic development. A recent study conducted by Metcalfe et al. (2010) tried to study the possible differences in SoA across lifespans. The authors compared children, young adults, and older participants’ performance using a computer game in which the task was to click on Xs and avoid Os. At times, the game included random distortions that decreased control. The participants had to judge how in control they felt and to rate their accuracy. The results showed that young adults were the most sensitive to discrepancies in control over their actions, as well as demonstrating their awareness of whether they were in control, compared to both children (8–10 years old) and older adults (mean age 75). Overall, these results showed that the metacognition of agency changes across the lifespan, suggesting a possible developmental trend. Although being the first to investigate age-related differences in SoA, this study used an explicit agency task, which may be influenced by different biases, such as prior expectations and beliefs about the task (Gawronski et al., 2007); thus, it says very little about the experience of agency, since it does not reflect or capture the feeling of agency that accompanies normal voluntary action (Synofzik et al., 2013).

In addition to Metcalfe et al.’s study, other studies have tried to investigate the emergence of agency. On one hand, studies focusing on the sense of the body (body awareness—for a review, see Rochat, 2010) and on the phenomenon of action–effect learning (Elsner and Aschersleben, 2003; Eenshuistra et al., 2004; Hauf et al., 2004; Elsner, 2007) show that: (i) the sense of body is already present in the first few months of life. Infants can therefore be considered agents in the world because they begin to gain control of their bodies and move voluntarily in the environment. In addition; (ii) action-effect learning seems to emerge even before the first year of age (Verschoor et al., 2010). On the other hand, other studies have shown that only 5-year-old children can report a mature experience of agency (Shultz et al., 1980; Astington, 2001; Lang and Perner, 2002). For example, Shultz et al. demonstrated that 5-year-old children are able to distinguish between a voluntary movement of the leg and a knee-jerk reflex However, all of the aforementioned studies—although aimed at studying the emergence of agency—are characterized by two important limits: (i) they contradict the fact that volition, which is strictly linked to the concept of agency, matures late during an individual’s development (Haggard, 2008), when the brain, in particular the frontal areas, reaches its full maturation (Giedd et al., 1999; Sowell et al., 1999); and (ii) they focus on low-level processes implicated in agency that are considered to be necessary conditions for the appearance of goal-directed behavior and action control, but are not sufficient to explain SoA’s complexity, which is rather a more sophisticated process. It includes in fact the ability to plan and control actions (planning, for example, to do something), but also the ability to identify actions’ consequences in the external world inhibiting erroneous behavior. SoA is therefore linked to the concept of responsibility (Moll et al., 2007; Frith, 2013, 2014): we are aware and responsible of what our actions produce. If, for example, I fight with someone and decide to voluntarily hit him/her, I am aware of the consequences that my action could produce (e.g., this person could fall down and injure himself, and I am aware of this). However, if the agent is a child, this feeling of being responsible for action consequences may not emerge in the same way. Below a certain age, children are not considered responsible for their actions: the minimum age of responsibility is the topic of important legal debates and varies from 7–18 years old (Frith, 2013, 2014). The general idea is that children may not be considered to be fully responsible for their own actions—and consequently not complete “agents”—since their frontal lobes are not fully matured yet (Moll et al., 2007; Mackintosh, 2011; Frith, 2013, 2014). In this sense it could be interesting to know how and when SoA develop.

The general purpose of the present work is therefore to understand how IB, as an implicit measure of SoA, can develop in children, by corroborating the existing literature, going beyond the basic aspects of agency, and overcoming the limits of the verbal reports that characterize the explicit level of SoA. If this background feeling of agency is innate, we could expect the same pattern to be found in young adults, or rather the temporal compression between voluntary action and sensory effects; otherwise, if IB is something that we acquire during our development, we could expect some differences between young adults and children.

The present study consists of two main experiments. In the first experiment (Experiment I), we sought to develop a new paradigm in order to assess IB at the implicit level. This purpose stems from the fact that the majority of studies uses either (i) the rotating spot method used by Libet et al. in 1983 (Libet et al., 1983; Haggard et al., 2002; Haggard and Clark, 2003; Haggard and Cole, 2007) or (ii) direct numerical judgments of the time interval between an action and its effect (Engbert et al., 2007, 2008; Cravo et al., 2009; Humphreys and Buehner, 2009). However, these approaches do not fit our case, since the rotating clock method could raise some problems with children, given the fact that the acquisition of both clock and time knowledge changes and improves with age (Vakali, 1991). In addition, time interval paradigms do not allow for the separate measurement of action binding (i.e., the shift of the action towards the effect) and effect binding (i.e., the shift of the effect towards the action), which seem to rely on different neural mechanisms (Moore et al., 2010; Wolpe et al., 2013). Therefore, the aim of Experiment I was to replicate the IB effect in a group of young adults using a new and more suitable paradigm, in order to test it later in children (Experiment II). We considered the method developed by Soon et al. (2008) to study the brain processes associated with the preparation of intentional actions as a reference point using a stream of letters. In this way, both the problem of the predictability of numbers using a clock and the problem of inaccuracy in time judgments, which can occur with rotating stimuli (van de Grind, 2002), can be avoided. In the second experiment (Experiment II), we tested IB in a group of 10-year-old children in order to investigate whether such an effect has already emerged at this age.

## Experiment I

The aim of Experiment I was twofold: (i) to create a paradigm suitable to test IB in children; and (ii) to test this paradigm in a group of young adults in order to verify the possibility of replicating the IB effect. In the case of replicating the IB effect in adults, the same paradigm would be adopted to test the IB effect in children in Experiment II.

### Method

#### Participants

Twenty participants (16 females; mean age in years: 23, SD: 1.41; education in years: 16.6, SD: 0.94) took part in the study. All participants were right-handed, as measured by the Edinburgh Handedness Inventory (Oldfield, 1971), had normal or corrected-to-normal vision, and lacked neurological and psychiatric pathologies. The study was conceived according to the Declaration of Helsinki and was approved by the Ethics Committee of the University of Padua. All participants gave their informed, written consent to participate in the study.

#### Apparatus and procedure

**Figure 1:**
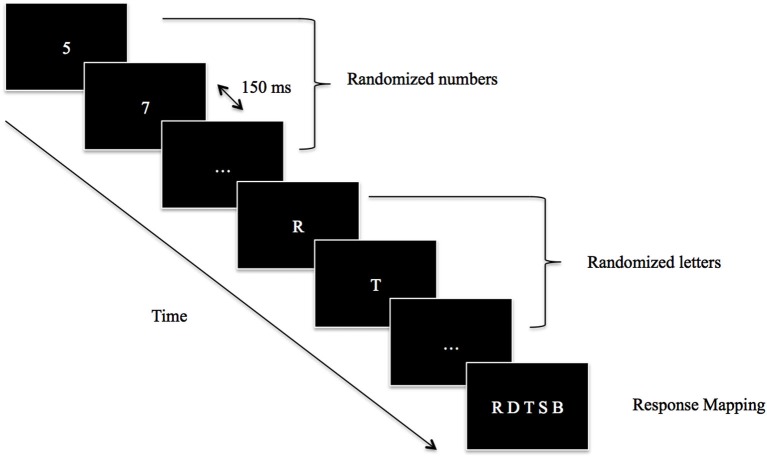
**Schematic illustration of the task structure**. Participants passively observed a stream of numbers and letters that was updated every 150 ms. The frame with “…” represented here the continuous flow either of numbers or letters. After the appearance of the event of interest (Voluntary Action, Involuntary Action, Tone 1, Tone 2) a response mapping appeared and participants chose the letter that was on the screen at the occurrence of the event of interest (e.g., Voluntary Action, Involuntary Action, Tone 1, Tone 2).

The experiment took place in a dimly illuminated room. The stimuli were presented on a 17-inch monitor controlled by a Pentium four PC programmed with E-Prime two (Psychology Software Tools, Pittsburgh, PA). The participants were seated comfortably in a chair at a viewing distance of 60 cm from the monitor. They were asked to passively observe a stream of unpredictable white, capital consonants at the center of a black screen. In order to prevent the participants from responding immediately after the occurrence of the letters, a series of randomized white numbers was displayed before the letters’ presentation (Figure 1). Each number and letter was presented separately and lasted for 150 ms, without time gaps in between. At the end of each trial, a set of response options (called “response mapping”) appeared on the screen. Five letters were presented on the screen, including the target letter (i.e., the letter that was on the screen at the actual appearance of the event of interest). After each trial, the participants had to choose the correct consonant using the keyboard with their left hand. We decided to introduce “response mapping” in order to avoid the significant involvement of a memory retrieval component in the task.

The experiment consisted of 4 baseline conditions (BCs) and 6 experimental conditions (ECs), for a total of 10 conditions (Table 1).

**Table 1:** **Conditions (Baseline and Experimental) and event judged by the participants in each condition**.

Condition	Event judged
**Baseline Conditions**	
1) Voluntary Action	Voluntary Action
2) Involuntary Action	Involuntary Action
3) Baseline Tone (Tone 1)	Baseline Tone (Tone 1)
4) Control Tone (Tone 2)	Control Tone (Tone 2)
**Experimental Conditions**	
5) Voluntary Action—250 ms—Tone 1	Voluntary Action
6) Voluntary Action—250 ms—Tone 1	Tone 1
7) Involuntary Action—250 ms—Tone 1	Involuntary Action
8) Involuntary Action—250 ms—Tone 1	Tone 1
9) Control Tone (Tone 2)—250 ms—Tone 1	Control Tone (Tone 2)
10) Control Tone (Tone 2)—250 ms—Tone 1	Tone 1

*Among the baseline conditions, only one event occurred per condition (e.g., voluntary action, involuntary action, Tone 1, Tone 2). For the experimental conditions, two events occurred per condition. The time interval between the first event (the voluntary action, the involuntary action, or Tone 2) and the second event (Tone 1) was set at 250 ms*.

**Figure 2:**
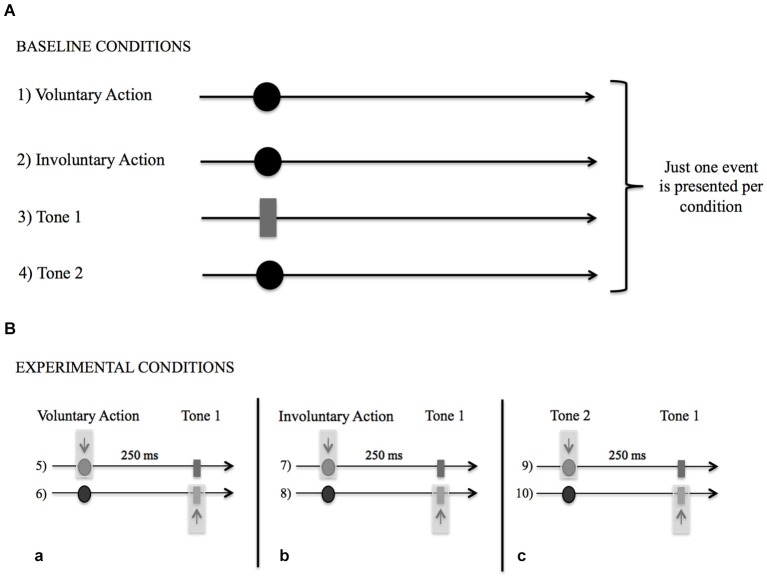
**(A)** Schematic representation of the Baseline Conditions (BCs) in which only one event (Voluntary Action, Involuntary Action, Tone 1, Tone 2) occurred per condition. While viewing the stream of numbers and letters participants had to remember which consonant was on the screen when: (1) they made a voluntary key-press; (2) they felt their right index finger moved down passively; (3) they heard Tone 1; and (4) they heard Tone 2. **(B)** Schematic representation of Experimental Conditions (ECs). **(a)** Participants judged the letter that was on the screen either when they made the Voluntary Action (5) or heard the Tone 1 (6). **(b)** Participants judged the letter that was on the screen either when they felt their right index finger moved down passively (Involuntary Action, 7) or heard the Tone 1 (8). **(c)** Participants judged the letter that was on the screen either when they heard the Tone 2 (9) or the Tone 1 (10).

Among the BCs (Figure 2A), only one event among voluntary action, involuntary action, Tone 1, or Tone 2 occurred per condition. The participants had to remember which consonant was on the screen when (1) they made a free voluntary key-press with their right index finger (acting as a baseline for voluntary action condition); (2) they felt their right index finger being passively moved down by a mechanical device (acting as a baseline for involuntary action condition); (3) they heard an auditory stimulus presented through headphones (1,000 Hz, 100-ms duration; baseline for tone condition: Tone 1); or (4) they heard another auditory control stimulus presented by headphones (same duration as Tone 1 but with a different pitch; baseline for tone control condition, Tone 2). In Condition (1), the participants had to wait until the letters’ appearance before responding, in order to avoid response anticipation (i.e., a key-press performed immediately after the trial onset). In Condition (2), a mechanical device was applied to the right index finger of the participants. The device was connected and activated by computer at a random interval after the trial’s onset. When the computer gave the input, the key and, consequently, the right index finger moved down, giving the participant the same physical perception as the voluntary key-press.

For the ECs, two events occurred per condition (Figure 2B). The participants had to judge (5) the onset of the voluntary action that produced Tone 1; (6) the onset of Tone 1 caused by the voluntary action; (7) the onset of the involuntary action that was followed by Tone 1; (8) the onset of Tone 1 activated by the involuntary action; (9) the onset of Tone 2 followed by Tone 1; (10) the onset of Tone 1 when activated by Tone 2. The time interval between the first event (the voluntary action, the involuntary action, or Tone 2) and the second event (Tone 1) was set at 250 ms.

Conditions involving the “involuntary action” and “Tone 2” were introduced as control conditions, in order to exclude the possible presence of IB in such conditions and investigate whether the results obtained for the voluntary action with the new paradigm were specific to SoA.

In all of the conditions, the stimuli were presented randomly, between 3 and 8 s after the trial onset. The stream of letters stopped randomly between 1.5 and 5 s after the event of interest. Thirty-three trials per condition were administered, for a total of 330 trials. The first three trials of each condition were discarded to allow for familiarization and were not included in the analysis. Each participant performed all of the conditions (BCs and ECs) in a different, random order over a single session.

### Data analysis

For each trial, we first calculated a judgment error (JE), which is the difference between the actual time of occurrence of the judged event and the perceived time of its occurrence. A negative JE was interpreted as anticipatory awareness of events (the participants perceived the event happening *before* it really did), while a positive JE was interpreted as delayed awareness (the participants perceived the event happening *after* it really did). For each condition, a mean JE (mJE), including both negative and positive values, was obtained. We obtained a total of 10 mJEs, one for each condition. Since the numerical value of the mJE in a single condition is generally not informative and difficult to interpret, the differences between the mJE of an identical physical event in two different contexts (the BCs and ECs) were calculated (i.e., the perceptual shift) by subtracting the mJE of each event in the BC (voluntary action, involuntary action, Tone 1, or Tone 2) from the mJE of the same event in the EC. For example, the shift of the action towards the tone (i.e., action binding) was calculated by subtracting the mJE of the voluntary action in the BC from the mJE of the voluntary action in the EC, whereas the shift of the tone towards the action (i.e., tone binding) was found by subtracting the mJE of Tone 1 in the BC from the mJE of the same Tone 1 in the EC. Therefore, calculating the perceptual shifts was important to control for the cross-modal synchronization judgments, which differ widely across individuals. Finally, we also computed an overall binding measure (Haggard et al., 2002; Haggard and Clark, 2003; Engbert et al., 2008) by combining the first (i.e., the action binding) and the second event (i.e., the tone binding). By calculating 250 ms—(action binding–tone binding), the obtained value represents the perceived linkage between an action and an effect, and provides an implicit measure of SoA.

### Results

Table 2 summarizes the mJEs, perceptual shifts, and overall binding.

**Table 2:** **mJEs, perceptual shifts and overall binding in young adults (Experiment I)**.

	Event judged	mJE (ms) ± sd	Mean shift (ms) ± sd	Overall binding (ms) ± sd
**Baseline Conditions**					
1) Voluntary Action (VA)	VA	14.25 ± 61.54		
2) Involuntary Action (IA)	IA	61.25 ± 59.98		
3) Baseline Tone (Tone 1)	Tone 1	40.75 ± 46.15		
4) Control Tone (Tone 2)	Tone 2	40.5 ± 44.36		
**Experimental Conditions**				
5) Voluntary Action—Tone 1	VA	90 ± 71.49	75.75 ± 60.14	98.75 ± 108.97
6) Voluntary Action—Tone 1	Tone 1	−34.75 ± 71.83	−75.5 ± 81.59	
7) Involuntary Action—Tone 1	IA	86.75 ± 62.5	25.5 ± 63.18	208.5 ± 93.16
8) Involuntary Action—Tone 1	Tone 1	24.75 ± 84.78	−16 ± 92	
9) Control Tone (Tone 2)—Tone 1	Tone 2	23.5 ± 39.8	−17 ± 46.52	262.5 ± 109.6
10) Control Tone (Tone 2)—Tone 1	Tone 1	36.25 ± 70.2	−4.5 ± 86.75	

*In the Experimental Conditions the first event (voluntary action, involuntary action, Tone 2) is separated by the second event (Tone 1) by a fixed 250-ms interval*.

**Figure 3:**
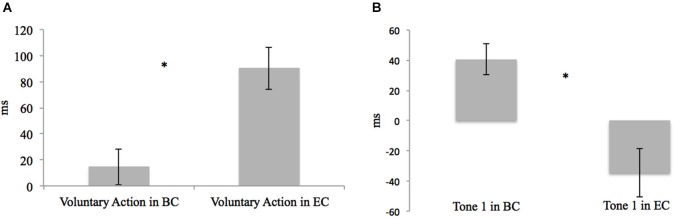
**(A)** Differences in the mJE of Voluntary Action in BC *vs*. EC in the young adult group. Error bars represent SEM and * indicates the significantly difference between BC and EC (*p* < 0.05). Here participants perceived the onset time of voluntary action later when it was followed by the tone (Voluntary Action in EC), as compared to the BC in which only the action was presented (Voluntary Action in BC). **(B)** Differences in mJE of Tone 1 in BC vs. EC in the young adult group. Error bars represent SEM and * indicates the significantly difference between BC and EC (*p* < 0.05). Here, participants perceived the onset time of the Tone 1 earlier when it was activated by the voluntary action (Tone 1 in EC), in comparison to the BC where only the tone was presented (Tone 1 in BC).

Using paired-sample *t*-tests, we first compared the mJE of a certain event in the BC with the mJE of the same event in the EC. For example, the mJE of a voluntary action in the BC was compared with the mJE of the voluntary action in the EC. Significant differences were only found in the context of voluntary action (voluntary action in the BC vs. voluntary action in the EC, *t*_19_ = −5.633, *p* < 0.001, and Tone 1 in the BC vs. Tone 1 in the EC, *t*_19_ = 4.138, *p* = 0.001) (Figure 3). Actions were therefore perceived later when followed by a tone, as compared to the BC, in which only the action was presented (Figure 3A). Differently, a tone was perceived earlier when it was activated by the action, in comparison to a BC where only the tone was presented (Figure 3B).

**Figure 4:**
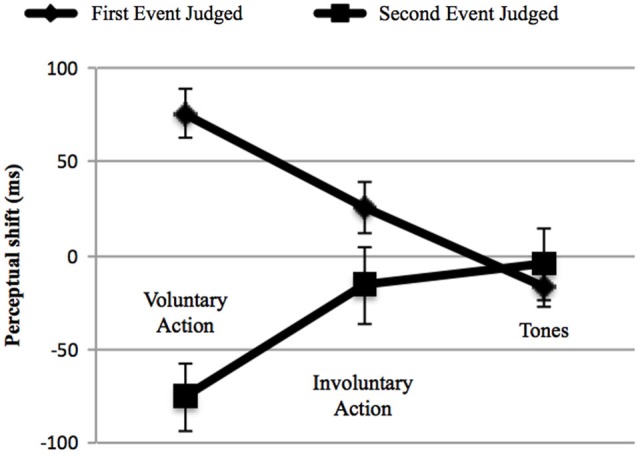
**Adults’ perceptual shifts in the three main contexts: Voluntary Action, Involuntary Action and Tones (i.e., the two auditory stimuli context: Tone 2–Tone 1)**. Error bars represent SEM. The first event judged (♦) could be either the voluntary action, the involuntary action or the Tone 2. The second event judged (■) was always represented by Tone 1. Negative perceptual shifts indicate than an event is perceived earlier in an experimental condition than in the baseline condition; positive perceptual shifts indicate that an event is perceived later in an experimental condition than in the baseline condition. Only voluntary actions produce IB (left). On the middle and on the right the involuntary action and two tones contexts are represented respectively, showing no IB.

In order to control for cross-modal synchronization judgments, we then calculated perceptual shifts using a 3 (“type of context”: voluntary, involuntary, and Tone 2) × 2 (“event judged”: either the first or the second) repeated-measures ANOVA. First, no main effect of action type was found, *F*_(2,38)_ = 0.782, *p* = 0.465, $\eta_{p}^{2}$ = 0.040, while the effect of the “event judged” was significant, *F*_(1,19)_ = 10.978, *p* = 0.004, $\eta_{p}^{2}$ = 0.366, with a shift of the first event towards the second (28.09 ms) and vice versa (−32 ms). In addition, a significant interaction between these two factors emerged, *F*_(2,38)_ = 21.697, *p* < 0.001; $\eta_{p}^{2}$ = 0.533 (Figure 4). We thus conducted a *post-hoc* analysis applying Bonferroni correction for multiple comparisons, in order to examine the interaction in more detail. The *post-hoc* analysis revealed that the difference between the first and the second event judged was only significant in the case of voluntary action (*p* < 0.001). In addition, concerning the first event judged, a significant difference was found for voluntary action, in comparison with involuntary action (*p* = 0.004) and Tone 2 (*p* < 0.001). Involuntary action and Tone 2 were also significantly different (*p* = 0.041). Significant differences also emerged when comparing the second event judged (e.g., Tone 1) (“voluntary action context” vs. “involuntary action context”, *p* = 0.035; “voluntary action context” vs. “two auditory stimuli context”, *p* = 0.002). Such interactions occurred because voluntary actions lead to a perceptual shift of action towards tone and vice versa, whereas this effect was reduced for the involuntary action context and for the two auditory stimuli context.

The repeated-measures ANOVA found a significant effect of the overall binding (i.e., the perceived linkage between action and effect), *F*_(2,38)_ = 21.697, *p* < 0.001, $\eta_{p}^{2}$ = 0.533. *Post-hoc* comparisons showed a significant difference in both the voluntary and involuntary contexts (*p* < 0.001). In addition, the “voluntary context” and the “two auditory stimuli context” (*p* < 0.001) were also significantly different. No significant differences were found between the “involuntary context” and the “two auditory stimuli context” (*p* = 0.205).

In summary, temporal compression (IB effect) was only evident in the context of voluntary action. The overall binding data indicate that the participants perceived the interval between their action and its effect as significantly shorter than it really was, although no direct judgment of the time interval’s duration was requested. Overall, our results revealed that, when participants were actively causing the beep (Tone 1), which was always presented 250 ms after their voluntary action, the onset of the voluntary action was perceived as occurring later, as if the action was “attracted” towards the tone. Analogously, the tone onset was perceived as “bound” to a voluntary action. This temporal compression phenomenon was only present in the case of voluntary action; when the beep followed the involuntary action or another control beep (Tone 2), such compression did not occur.

Using a new methodology, we replicated the IB effect and therefore proceeded to test IB in children (see Experiment II).

## Experiment II

Given the positive results of Experiment I, we decided to use the new paradigm validated in Experiment I in order to test IB in children.

### Method

#### Participants

Eighteen participants (14 females; mean age in years: 10, SD: 0.97; education in years: 5.05, SD: 0.87) took part in the study. All participants were right-handed, as measured by the Edinburgh Handedness Questionnaire (Oldfield, 1971), had normal or corrected to-normal vision, and lacked neurological and psychiatric pathologies. The study was approved by the Ethics Committee of the University of Padua and was conducted according to the Declaration of Helsinki. Informed consent was obtained from parents.

#### Apparatus and procedure

The apparatus and the procedure were the same as those used in Experiment I. In addition, the participants received basic neuropsychological screenings in order to exclude children with cognitive problems, which could interfere with the task. The tests included the Colored Progressive Matrices (Pruneti et al., 1996), the Trial Making Test (TMT; forms A, AB, and B—Scarpa et al., 2006), and the Bells Test (Biancardi and Stoppa, 1997).

### Results I: IB in children

All participants had an IQ above 100 and obtained normal scores on the TMT and Bells Test. Table 3 presents their mJEs, perceptual shifts, and overall binding.

**Table 3:** **mJEs, perceptual shifts and overall binding in children (Experiment II)**.

	Event judged	mJE (ms) ± sd	Mean shift (ms) ± sd	Overall binding (ms) ± sd
**Baseline Conditions**				
1) Voluntary Action (VA)	VA	−19.72 ± 69.82		
2) Involuntary Action (IA)	IA	81.39 ± 47.95		
3) Baseline Tone (Tone 1)	Tone 1	79.17 ± 23.28		
4) Control Tone (Tone 2)	Tone 2	82.22 ± 49.44		
**Experimental Conditions**				
5) Voluntary Action—Tone 1	VA	1.67 ± 74.2	21.39 ± 72.86	169.17 ± 101.65
6) Voluntary Action—Tone 1	Tone 1	19.72 ± 67.11	−59.44 ± 64.39	
7) Involuntary Action—Tone 1	IA	72.5 ± 61.05	−8.89 ± 44.73	209.72 ± 93.23
8) Involuntary Action—Tone 1	Tone 1	30 ± 62.47	−49.17 ± 61.29	
9) Control Tone (Tone 2)—Tone 1	Tone 2	81.66 ± 33.91	−0.56 ± 52.07	208.33 ± 72.58
10) Control Tone (Tone 2)—Tone 1	Tone 1	36.94 ± 55.18	−42.22 ± 51.63	

*In the Experimental Conditions the first event (voluntary action, involuntary action, Tone 2) is separated by the second event (Tone 1) by a fixed 250-ms interval*.

We compared the mJE of each event in the BC with the mJE of the same event in the EC using paired-samples *t*-tests. Significant differences were only found in the perception of Tone 1 in the EC compared to the BC, in which the tone was presented alone. However, these differences were not limited to the case of the voluntary action (*t*_17_ = 3.916, *p* = 0.001) like in adults; they also extended to the case of the two control conditions: involuntary action (*t*_17_ = 3.403, *p* = 0.003) and Tone 2 (*t*_17_ = 3.470, *p* = 0.003). Tone 1 (i.e., the effect/beep) was therefore perceived earlier when it followed the voluntary action, the involuntary action, or Tone 2, as compared to the BC.

**Figure 5:**
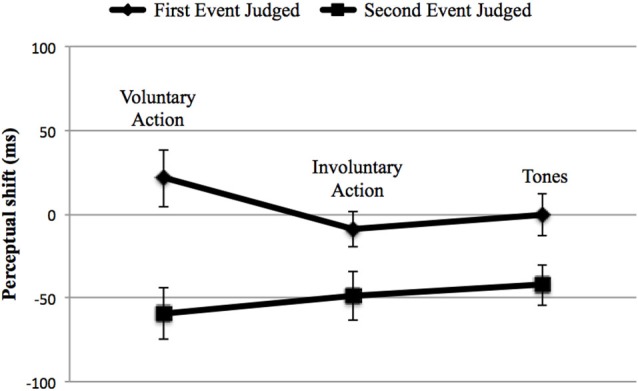
**Children’ perceptual shifts in the three main context: Voluntary Action, Involuntary Action and Tones (i.e., the two auditory stimuli context: Tone 2–Tone 1)**. Error bars represent SEM. The first event judged (♦) could be either the voluntary action, the involuntary action or the Tone 2. The second event judged (■) was always represented by Tone 1. Negative perceptual shifts indicate than an event is perceived earlier in an experimental condition than in the baseline condition; positive perceptual shifts indicate that an event is perceived later in an experimental condition than in the baseline condition. No temporal compression occurred for the voluntary action.

We also analyzed the perceptual shifts in order to investigate IB, as in Experiment 1. The repeated-measures ANOVA revealed no effect of action type, *F*_(2,34)_ = 0.341, *p* = 0.713, $\eta_{p}^{2}$ = 0.020, except for a main effect of the event judged (*F*_(1,17)_ = 18.03, *p* = 0.001, $\eta_{p}^{2}$ = 0.515) having a larger shift for the second event towards the first one (−50.28 ms vs. 3.98 ms). The interaction between the two factors was not significant, *F*_(2,34)_ = 1.233, *p* = 0.304, $\eta_{p}^{2}$ = 0.068 (Figure 5), indicating that no temporal compression occurred for the voluntary action.

When considering the overall binding, no differences were found between the three contexts (“voluntary action”, “involuntary action”, and the “two auditory stimuli context”), *F*_(2,34)_ = 1.233, *p* = 0.304, $\eta_{p}^{2}$ = 0.068.

The results showed that no IB was present in the 10-year-old children. Although a sort of minimal temporal compression seems to exist in the case of voluntary action, it does not reach significance, when compared to the two control conditions.

### Results II: between-group comparisons

In order to better understand the lack of IB in children, we then proceeded to compare the degree of binding between the two groups. Concerning BCs, no differences were found in the “voluntary action condition”, *t*_36_ = 1.594, *p* = 0.120, or in the “involuntary action condition”, *t*_36_ = −1.135, *p* = 0.264. However, significant differences were found in the case of Tone 1, *t*_36_ = −3.287, *p* = 0.003, and Tone 2, *t*_36_ = −2.742, *p* = 0.009. In our study, adults perceived tones better than children.

Concerning ECs, on the other hand, significant differences were found in the perception of the voluntary action during the EC, *t*_36_ = 3,736, *p* = 0.001, as well as of Tone 1 following the voluntary action, *t*_36_ = −2.408, *p* = 0.021, and of Tone 2 in the EC, *t*_36_ = −4.821, *p* < 0.001.

The baseline differences in the perception of tones can explain the differences shown in the perception of Tone 1 following the voluntary action and Tone 2 in the EC, but they cannot account for the differences found in the case of the voluntary action. While the adults perceived voluntary actions significantly later (towards the tone) compared to the BC, in children, although the direction of the shift was opposite between BC (−19.72 ms) and EC (+1.67 ms), such changes did not reach a significant level. We therefore analyzed the perceptual shifts using 3 × 2 repeated measures ANOVA, using the group (children vs. young adults) as the between-factor. First, we did not find a significant main effect of group, *F*_(1,36)_ = 4.012, *p* = 0.053, $\eta_{p}^{2}$ = 0.100. A predicted and highly significant main effect of the judged event was observed, *F*_(1,36)_ = 25.490, *p* < 0.001, $\eta_{p}^{2}$ = 0.415, with the first event showing a delayed shift towards the second (16.03 ms) and vice versa (−41.14 ms). Most importantly, the interaction between group, type of action (voluntary, involuntary, and Tone 2), and judged event (first vs. second) was significant, *F*_(2.72)_ = 5.242, *p* = 0.007, $\eta_{p}^{2}$ = 0.127. The only significant difference between the two groups emerged in the case of the action-binding effect (i.e., the shift of the voluntary action towards the tone) (*p* = 0.016). No significant differences were found between the shifts in the other control contexts.

**Figure 6:**
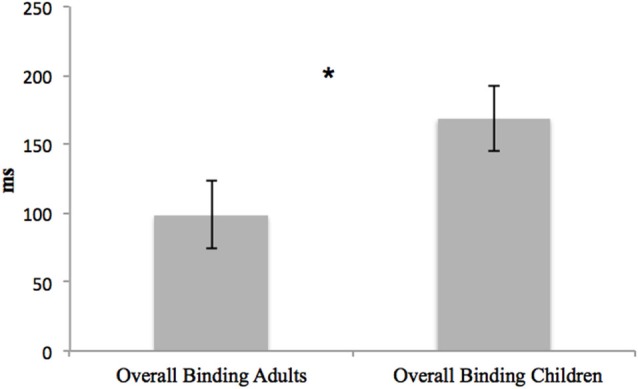
**Differences in the voluntary action overall binding between young adults and children**. Error bars represent SEM and * indicates the significantly difference in overall binding between the two groups. Only adults present IB effect, showing temporal compression between voluntary action and its effect.

Also, the overall bindings were compared between the two groups. No main effect of group, *F*_(1,36)_ = 0.066, *p* = 0.799, $\eta_{p}^{2}$ = 0.002, was found, but a main effect of overall binding emerged, *F*_(2,72)_ = 14.92, *p* < 0.001, $\eta_{p}^{2}$ = 0.293: temporal compression was only present in the voluntary action context (*p* < 0.001). A significant interaction between overall binding and group emerged, *F*_(2,72)_ = 5.242, *p* = 0.007, $\eta_{p}^{2}$ = 0.127. Children and young adults only differed in the case of the “voluntary action context” (*p* = 0.047) (Figure 6).

To summarize, the only significant difference between adults and children regarded the “voluntary action context”, in particular, the shift of the action towards the tone. No differences emerged in the case of the two control contexts. These data are important for explaining the lack of IB effect in children.

## General discussion

The aim of the present study was to investigate the ontogenetic development of IB as an implicit measure of SoA, by taking advantage of its superiority over explicit tasks (verbal self-reports) (Wolpe and Rowe, 2014).

In Experiment I, a new, reliable paradigm for assessing IB was introduced and tested in a group of young adults. The results showed that only voluntary actions were perceived as occurring later in time than they really were (e.g., as more adjacent to the following tone in temporal terms); on the other hand, tones were perceived as occurring earlier than they really were (e.g., closer to actions in time). Such temporal compression was limited to the context of voluntary conditions. We considered these results as a proof of the IB effect.

In Experiment II, we tested the same paradigm considered in Experiment I in children. The results showed a reduction of IB, both in the context of “voluntary action” and in the two control conditions (“involuntary action” and “tones”). This lack of findings could be explained within the frame of the “warning-signal hypothesis” (Droit-Volet, 2003, 2011), which demonstrates that, when target stimuli are preceded by warning signals, the amount of time required for stimulus processing decreases and accuracy improves. In fact, when the children had to evaluate the second event in the ECs (e.g., Tone 1), judgment accuracy significantly increased in comparison to the BC, in which only Tone 1 was presented. In fact, in the BC conditions, children perceived Tone 1 after its real onset; when Tone 1 was activated by the voluntary action, it was perceived more accurately. The same pattern also emerged when Tone 1 followed the involuntary action and Tone 2. We therefore speculated that children could consider the first event (voluntary action, involuntary action, or Tone 2) to be a warning signal for the arrival of the subsequent tone. The warning-signal hypothesis found confirmation in developmental studies showing that a warning event can actually act as an attentional preparation cue and then lead to performance improvements (Droit-Volet, 2003, 2011). In fact, children are more accurate in judging the second event in the ECs compared to the BC, in which only one event is presented at random latencies. On the other hand, when an evaluation of the first event in the ECs is requested, no significant differences emerged, in comparison to the BCs. In this case, the children did not seem to consider the effect (e.g., Tone 1) following the voluntary action, the involuntary action, or Tone 2, and only focused their attention on the first event.

Another possible explanation that is worth taking into account refers to the “lack of inhibitory control”, which is common in children. Several classic developmental studies have demonstrated that the ability to suppress irrelevant information becomes more efficient with age (Diamond and Doar, 1989; Durston et al., 2002). As a matter of fact, performance on Stroop, flanker, and go/no-go tasks continues to develop over childhood and does not reach its maximum until 12 years of age or later (Carver et al., 2001; Bunge et al., 2002; Durston et al., 2002). In our study, the children could have more accurately judged the onset of the second event in the ECs compared to the BCs because they were influenced by the presence of the first event, not because they treated the first event as a warning stimulus (warning signal theory: Droit-Volet, 2003, 2011). In fact, when Tone 1 was presented alone in the BC, it was perceived 79.17 ms after its real appearance. When it was activated by the first event in the ECs (voluntary action, involuntary action, or Tone 2), Tone 1 was perceived earlier and, consequently, more accurately, compared to the BC. When the children had to evaluate the second event in the ECs, they were not able to disengage their attention from the irrelevant stimulus (i.e., the first event), which was therefore not well-inhibited. For this reason, the second event was perceived earlier and consequently more accurately, compared to the BC.

Summarizing both hypotheses (the warning signal and the lack of inhibitory control) could represent a plausible explanation for our results. However, the lack of an inhibitory control hypothesis could better fit our data: in fact, in order to control the cross-modal estimations in timing judgments, we have to consider the perceptual shifts, not just the difference between the BC and the EC. Figure 5 shows that the second event seems to be influenced by the first one: the effect (e.g., Tone 1) is perceived earlier towards the first event independently, by the context, and the shift is significantly different between the first and the second event, with a greater shift for the second one. It is therefore more likely that the children were unable to manage the interference caused by the first event and, consequently, to correctly evaluate the beep (e.g., Tone 1). Judging the second event correctly implies that attention has to be disengaged from the previously presented stimulus (i.e., the first event). This hypothesis finds confirmation in the literature from several studies reporting difficulties in suppressing activated, but irrelevant, information in children. In these cases, irrelevant information exploited resources that otherwise would be available to process relevant information, which led to global performance decreases (Tipper et al., 1989; Bjorklund and Harnishfeger, 1990; Rubia et al., 2000; Lorsbach and Reimer, 2011). A point worth mentioning is the fact that, in the case of the first event—in particular, the perception of the voluntary action—something different occurred compared to the two control conditions. Although this difference did not reach a significant level, it is worth underlining that the change in the case of voluntary action was greater in the BC (−19.72 ms) than in the EC (1.67 ms), When the children had to evaluate the consonant on the screen when they made the key-press in the BC, they perceived the onset of the voluntary action earlier than it really was. On the other hand, when the voluntary action caused the tone in the EC, the action was perceived later towards the tone, compared to the BC (1.67 ms). Also, the shift direction was different: in the BC, the voluntary action was perceived before it really occurred, while in the EC, it shifted towards the consequent tone. Such changes did not occur in the two control conditions. Therefore, it seems that a sort of temporal compression was developing in the children.

Considering the overall binding (i.e., the perceived linkage between action and effect), no differences emerged between the three different contexts (i.e., “the voluntary action context”, “the involuntary action context”, and “the two tones context”), although a sort of temporal compression seems to be present in the case of voluntary action. This lack of effect could be explained by looking at Droit-Volet’s (2013) and Droit-Volet et al.’s (2004, 2007). First, the children could have encountered difficulties with this task (as a result of their limited attentional control capacities; for a review, see Brainerd and Dempster, 1995), particularly with the stream of visual letters, since the dominance of audition over vision has been reported in the processing of time (for a review, see Pouthas et al., 1993). In fact, auditory stimuli could be captured more easily compared to visual stimuli because audition is more specialized for processing temporal information. The second aspect refers to timing sensitivity, which increases with age and is not completely present in 8-year-olds (for a review, see Droit-Volet et al., 2006).

In addition, when comparing the data obtained from the adults and the children, the overall binding pattern of results within the two groups appears to be different. The two groups did not differ in terms of control conditions; rather, they only showed significant differences in the “voluntary action condition”, suggesting that temporal compression only characterizes the adults’ performance (Figure 6). On the other hand, when considering action and effect binding separately, the two groups only exhibited differences concerning action binding (i.e., the shift of the action towards the tone). This result can be explained by considering the two different processes implicated in action-and-effect binding (Moore et al., 2010; Wolpe et al., 2013). Effect binding seems to rely on a pre-activation mechanism (Waszak et al., 2012); the neural representation of a sensory outcome following a voluntary action is activated before its occurrence. When the predicted sensory event occurs, the perceptual threshold is reached faster than when the event is not predicted. Consequently, estimation errors are smaller in the ECs than they are in the BCs, leading to effect binding. On the other hand, action binding depends on both predictive motor control and inferential processes (Moore and Haggard, 2008). It could be possible that the pre-activation mechanism is already fully efficient in children, while mechanisms implicated in action binding are still being developed.

In conclusion, our research represents a substantial contribution to the comprehension of SoA mechanisms. First, we replicated the IB effect with a new paradigm that could represent an alternative to both the Libet clock and the time interval methods, thus avoiding the problems related to rotating stimuli and disentangling action binding from effect-binding processes respectively. In this sense, it is crucial to better investigate the contribution of predictive (e.g., motor command signals: Wolpert and Ghahramani, 2000; Blakemore et al., 2001) and reconstructive processes (the integration of external sensory feedback: Wegner, 2002) in children by varying the conditional probabilities of the tones and actions (Moore and Haggard, 2008). Second, our data improve and corroborate results from the literature on the ontogenetic development of agency, while going beyond its basic aspects (body awareness and action–effect learning). The use of IB as an implicit measure of SoA implies that more complex cognitive abilities are considered (i.e., executive functions), thus better depicting the complexity of SoA. In this sense, the present study is the first attempt to investigate IB as an implicit measure of SoA, in a group of children using an implicit measure of it. We found reduced IB effects in children. In fact, although the patterns of the adults and the children regarding the “voluntary action context” seemed to be similar, the results obtained from the children seem to suggest a tendency to be more focused on voluntary action, without taking the effects produced by it into account. If we consider IB to be an “adaptive illusion” that gives us a strong sense of causality and helps us to consider ourselves as responsible for certain effects, such an illusion does not seem to deceive children, maybe because the necessary cognitive skills have not been acquired yet (i.e., inhibitory control or the ability to attend selectively to critical stimuli while ignoring irrelevant information). These cognitive abilities, which belong to the executive functions’ family, are generally connected with the functionality of frontal areas. Hence, it is possible that children may not possess IB because such areas, which are fundamental for the acquisition of the cognitive skills necessary to process IB, are not developed yet, like in adults. For all of these reasons, we suggest that IB may follow a developmental trend. It may be acquired gradually during ontogenesis, parallel with the maturation of the frontal cortical network. Since SoA and IB seem to share the same common cognitive mechanisms and neural networks (David et al., 2008; Moore et al., 2010; Moore and Obhi, 2012; Kühn et al., 2013; Wolpe et al., 2014), we could therefore speculate that, in conjunction with the reduction of IB, children also show diminished SoA, which does not allow them to understand the consequences of their actions. However, our results refer to IB, and speculations on SoA remain limited. The possible hypothesis of a link between reduced IB and the maturation of frontal areas in children remains an open issue that needs to be tested by means of neuroimaging techniques. Future studies are required to confirm our hypothesis, in order to provide a further step in the contextualization of SoA dynamics throughout age.

## Conflict of interest statement

The authors declare that the research was conducted in the absence of any commercial or financial relationships that could be construed as a potential conflict of interest.
